# Supplementary material for: Antibiotic Resistance/Susceptibility Profiles of Staphylococcus equorum Strains from Cheese, and Genome Analysis for Antibiotic Resistance Genes
Source: Int J Mol Sci. 2023 Jul 19;24(14):11657. doi: 10.3390/ijms241411657 (PMC10380560; doi:10.3390/ijms241411657)
Supplement: Supplementary file 1 [file ijms-24-11657-s001.zip › ijms-2462464-supplementary.pdf]

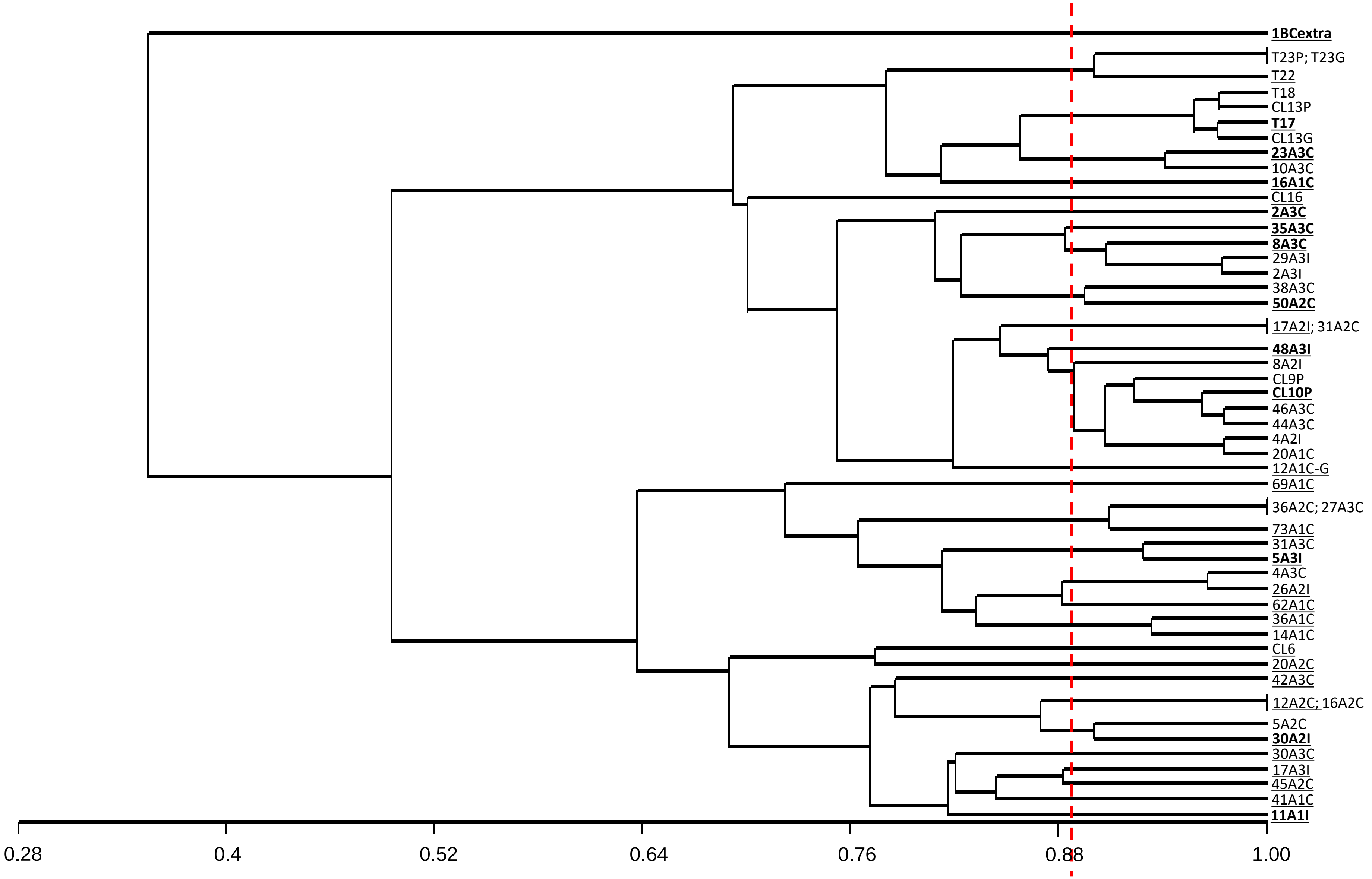

Figure S1.- Dendrogram of similarity obtained from the RAPD and rep-PCR typing profiles expressed by the Sorensen's coefficient (in %) for a total of 53 *Staphylococcus equorum* isolates from cheese. Clustering was performed by the unweighted pair group method using arithmetic averages (UPGMA). The dotted red line indicates the 0.90% similarity index considered to select different strains. Underlined strains subjected to antibiotic testing; in bold, sequenced strains.

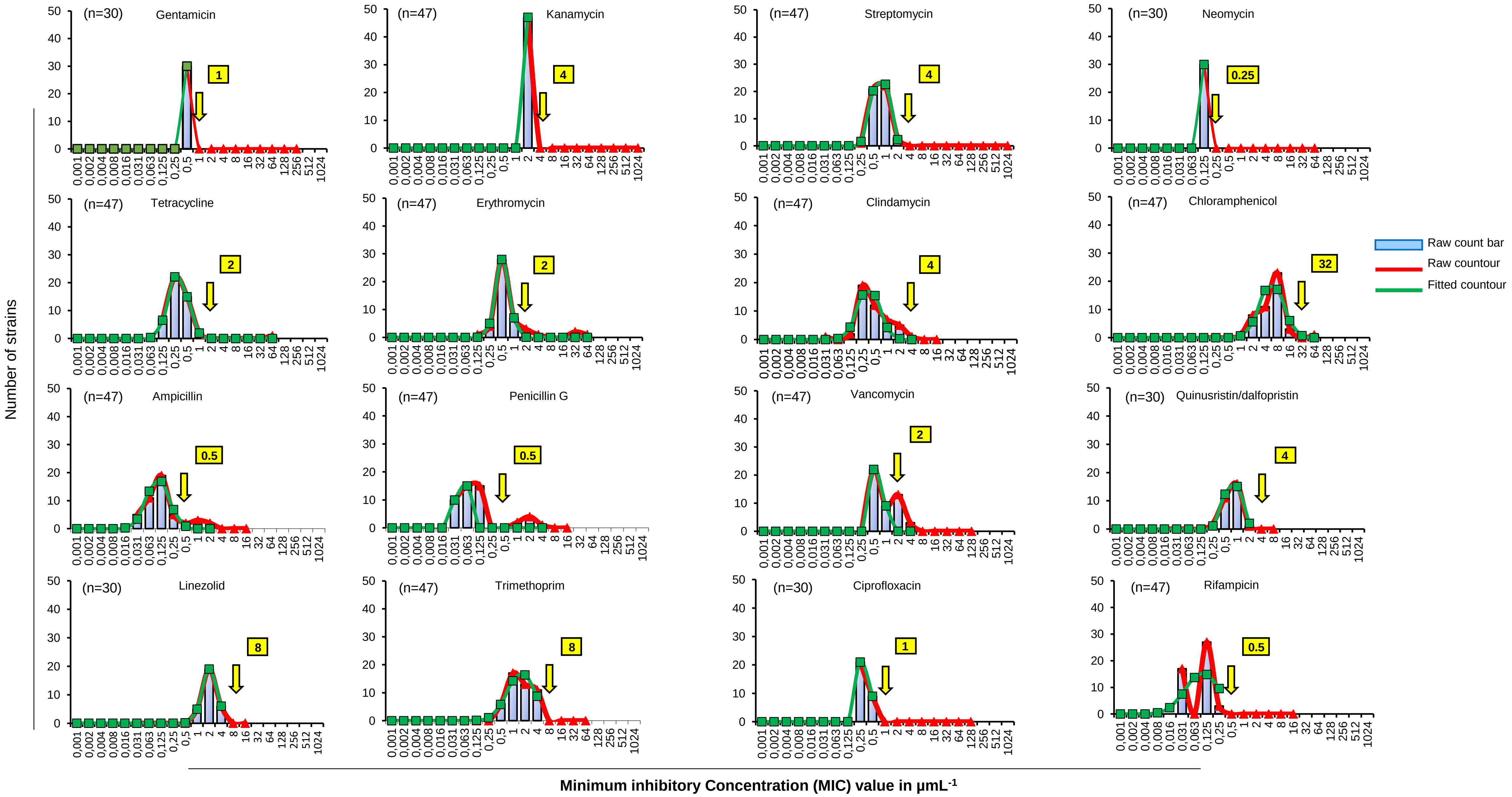

Figure S2: Non-linear regression fitting distribution of the MIC values of sixteen antibiotics to *Staphylococcus equorum* strains in this work and the values reported by Marty et al. [18] with ECOFFinder according to Turnidge et al. [43]. The yellow arrows indicate the suggested breaking points proposed in this study to separate susceptible from resistant *S. equorum* cheese strains. For gentamicin, neomycin, quinupristin-dalfopristin, linezolid, and ciprofloxacin only the values of this work were available (*n*=30).

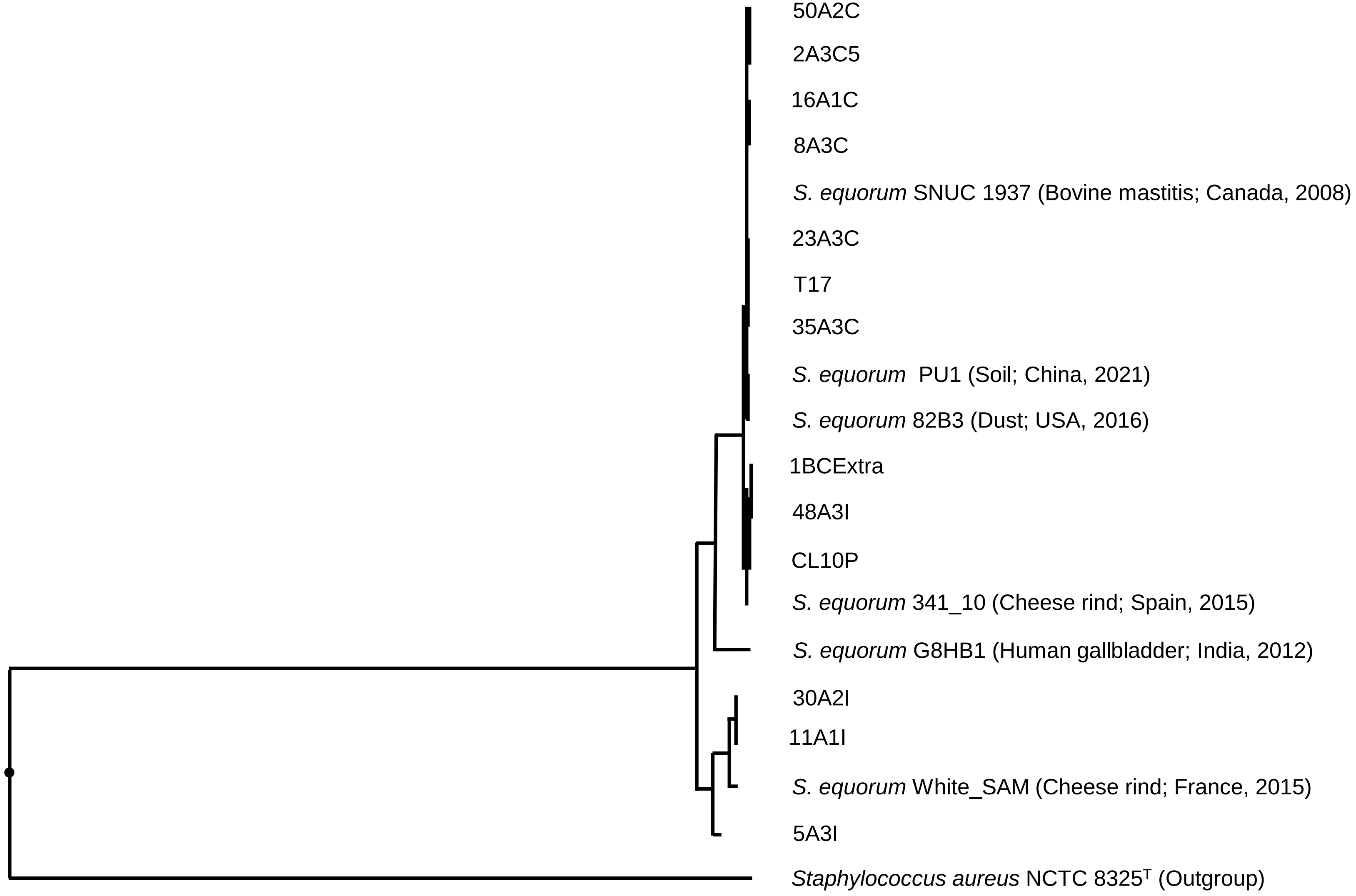

Figure S3.- Phylogenomic analysis of the *Staphylococcus equorum* strains from cheese and some others from databases isolated around the globe from different sources and in different periods carried out using 500 genes at the BV-BRC Server (https://www.bv.brc.org). The *Staphylococcus aureus* type strain NCTC 8325^T was used as an outgroup. The tree was rooted at the midpoint.

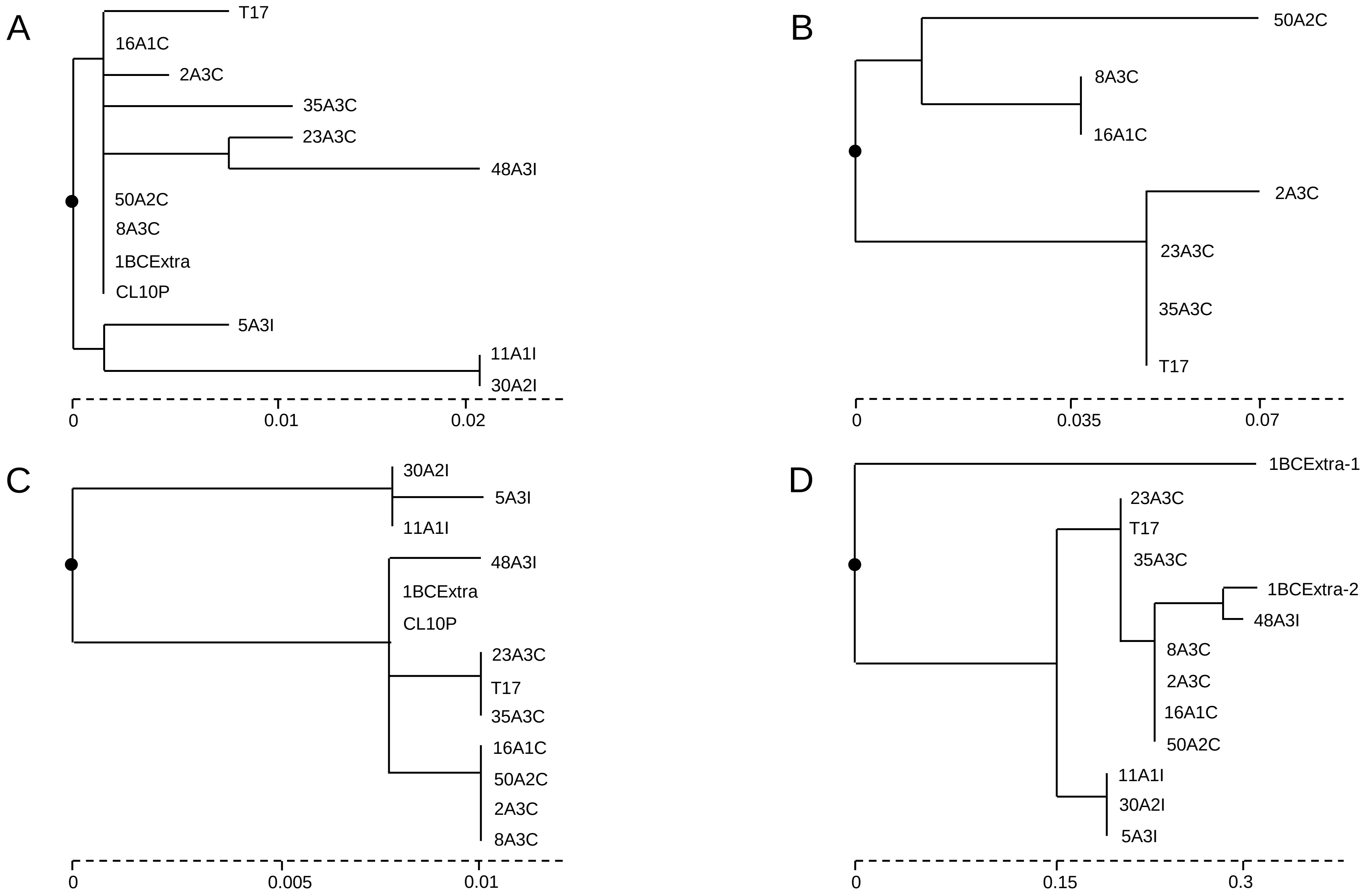

Figure S4. Phylogenetic trees of the proteins encoded by the antibiotic resistance genes *bla* (A), *msr*(A) (B), *norA* (C), and *fosB/fosD* (D). Alignment and phylogenetic reconstructions were performed using the function "build" of ETE3 3.1.2 as implemented on GenomeNet (https://www.genome.jp/tools/ete/; accessed on 6 April 2023). ML trees were inferred using PhyML v20160115 ran with model and parameters: -pinv e -alpha e -nclasses 4 -o tlr -f m -bootstrap 100.

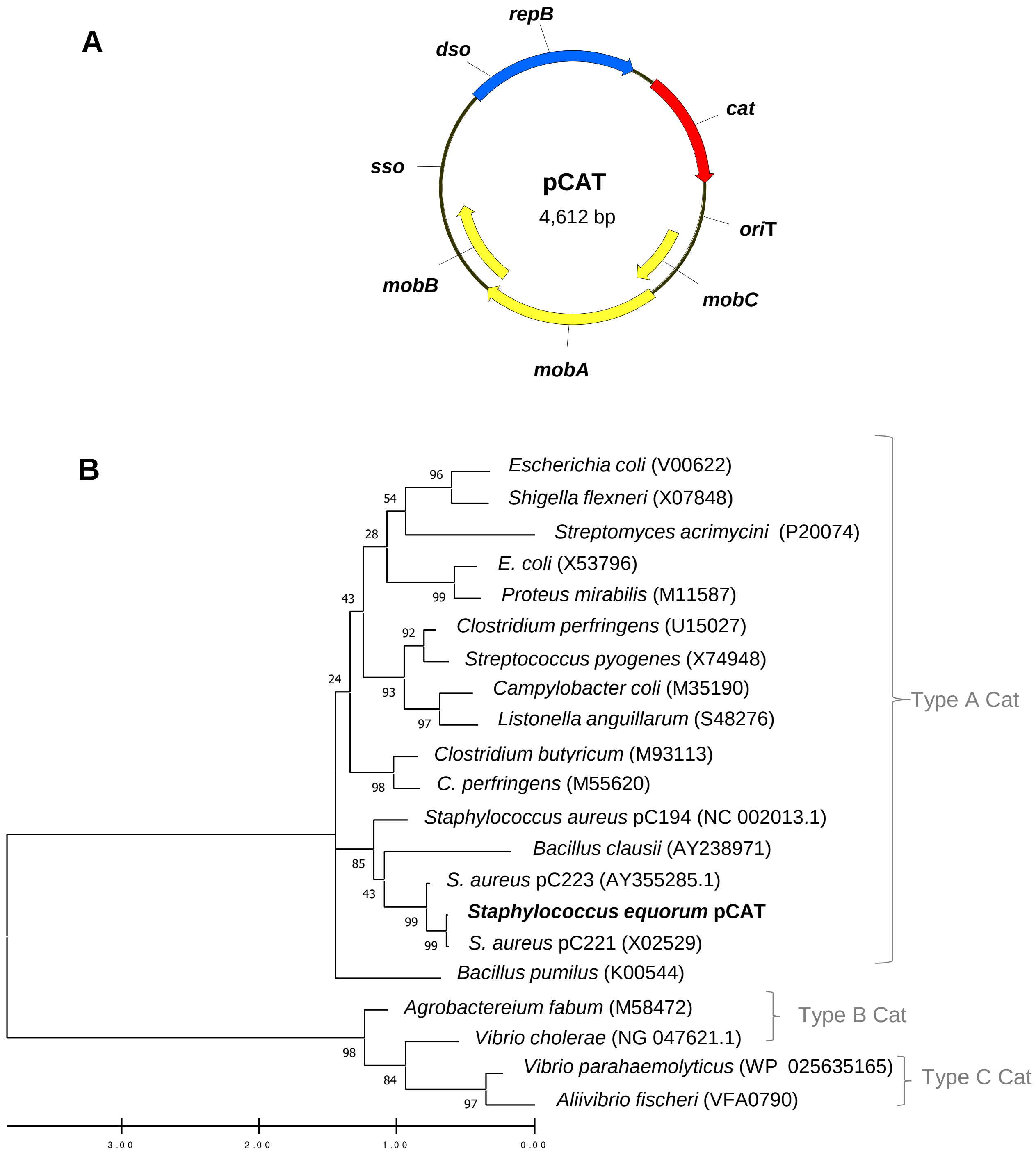

Figure S5.- Panel A, genetic map of plasmid pCAT from *S. equorum* 35A3C harbouring the *cat* gene encoding a type A-7 *o*-chloramphenicol acetyltransferase. Panel B, phylogenetic tree of the Cat protein from pCAT (in bold) and others from databases.

Table S1.- Minimum inhibitory concentration (MIC) values of 16 antibiotics to 30 *Staphylococcus equorum* strains isolated from cheese.

Strain	Antibiotic (MIC μg mL^{-1})[a]															
	**Gm**	**Km**	**Sm**	**Nm**	**Tc**	**Em**	**Cli**	**Cm**	**Am**	**PG**	**Van**	**Q-Da**	**Lnz**	**Tm**	**Cip**	**Rif**
CL6	≤0.5	≤2	1	≤0.12	0.25	0.5	0.5	8	0.12	0.12	0.5	0.5	2	1	≤0.25	≤0.12
**CL10P**	≤0.5	≤2	1	≤0.12	0.5	0.5	1	8	0.06	≤0.03	0.5	0.5	2	4	0.5	≤0.12
CL16	≤0.5	≤2	1	≤0.12	0.5	1	1	8	1[b]	2[b]	0.5	1	2	4	0.5	≤0.12
**T17**	≤0.5	≤2	0.5	≤0.12	0.5	4	2	16	0.12	0.12	0.5	1	4	2	≤0.25	≤0.12
T22	≤0.5	≤2	0.5	≤0.12	0.25	0.5	0.25	8	0.06	≤0.03	0.5	0.5	2	4	≤0.25	0.25
**1BCExtra**	≤0.5	≤2	1	≤0.12	0.25	1	0.5	8	≤0.03	0.06	0.5	0.25	2	2	≤0.25	≤0.12
**2A3C**	≤0.5	≤2	1	≤0.12	0.25	2	2	8	0.12	0.06	0.5	1	2	2	≤0.25	≤0.12
**5A3I**	≤0.5	≤2	1	≤0.12	1	1	2	8	0.12	0.12	1	1	4	4	≤0.25	≤0.12
**8A3C**	≤0.5	≤2	1	≤0.12	0.5	64[b]	2	8	0.06	0.12	0.5	1	2	2	≤0.25	≤0.12
**11A1I**	≤0.5	≤2	0.5	≤0.12	0.5	0.5	0.12	8	0.06	0.06	0.5	0.5	2	1	0.5	≤0.12
12A1C-G	≤0.5	≤2	1	≤0.12	0.5	0.5	1	8	≤0.03	0.06	0.5	1	2	2	≤0.25	≤0.12
12A2C	≤0.5	≤2	0.5	≤0.12	0.25	0.25	1	4	0.38[b]	2[b]	0.5	1	1	2	≤0.25	≤0.12
**16A1C**	≤0.5	≤2	2	≤0.12	1	32[b]	2	8	0.25	0.12	1	1	4	4	≤0.25	≤0.12
17A2I	≤0.5	≤2	1	≤0.12	0.25	1	0.25	8	0.12	0.12	1	0.5	2	4	≤0.25	≤0.12
17A3I	≤0.5	≤2	0.5	≤0.12	0.25	0.5	0.25	8	0.12	0.12	0.5	0.5	2	0.5	0.5	≤0.12
20A2C	≤0.5	≤2	1	≤0.12	0.25	0.5	0.25	8	0.06	0.12	0.5	0.5	2	1	0.5	≤0.12
**23A3C**	≤0.5	≤2	1	≤0.12	0.5	2	4	16	0.25	0.12	1	2	4	4	≤0.25	≤0.12
26A2I	≤0.5	≤2	0.5	≤0.12	0.5	1	0.5	8	0.75[b]	0.75[b]	0.5	1	2	1	≤0.25	≤0.12
**30A2I**	≤0.5	≤2	0.5	≤0.12	0.25	0.25	0.12	4	0.06	≤0.03	0.5	0.5	2	2	0.5	≤0.12
30A3C	≤0.5	≤2	0.5	≤0.12	0.25	0.5	0.25	8	0.75[b]	1.5[b]	1	0.5	1	1	0.5	≤0.12
**35A3C**	≤0.5	≤2	0.5	≤0.12	0.5	2	0.5	64	≤0.03	≤0.03	0.5	0.5	4	1	≤0.25	0.25
36A1C	≤0.5	≤2	1	≤0.12	0.5	0.5	1	8	0.12	0.12	1	1	2	4	≤0.25	≤0.12
41A1C	≤0.5	≤2	0.5	≤0.12	0.5	0.5	0.5	8	0.25	0.12	1	1	2	1	≤0.25	≤0.12
42A3C	≤0.5	≤2	0.5	≤0.12	0.25	0.5	0.25	8	1.5[b]	3[b]	0.5	0.5	1	1	0.5	≤0.12
45A2C	≤0.5	≤2	1	≤0.12	0.25	0.25	1	4	2[b]	1.5[b]	0.5	1	1	1	≤0.25	≤0.12
**48A3I**	≤0.5	≤2	2	≤0.12	0.5	0.5	0.25	16	0.06	≤0.03	1	0.25	2	4	0.5	≤0.12
**50A2C**	≤0.5	≤2	1	≤0.12	0.25	24[b]	0.5	8	0.06	≤0.03	0.5	1	1	1	≤0.25	≤0.12
62A1C	≤0.5	≤2	1	≤0.12	0.5	0.5	1	8	0.12	0.12	1	1	4	4	≤0.25	0.25
69A1C	≤0.5	≤2	1	≤0.12	0.25	0.5	0.25	8	≤0.03	≤0.03	0.5	1	2	2	≤0.25	≤0.12
73A1C	≤0.5	≤2	0.5	≤0.12	0.5	1	0.5	8	0.5[b]	1[b]	0.5	1	2	0.5	≤0.25	≤0.12
Breakpoints (μg mL^{-1})[c]																
**CLSI**	R ≥16	(-)	(-)	(-)	R ≥16	R ≥8	R ≥4	R ≥32	(-)	R ≥0.25	R ≥32	R ≥4	R ≥8	R ≥16	R ≥4	R ≥4
**EUCAST**	R ≥2	(-)	(-)	(-)	R ≥1	R ≥1	R ≥0.25	(-)	(-)	(-)	R ≥4	R ≥1	R ≥4	R ≥4	R ≥1	R ≥0.06

[a]Key of antibiotics: Gm, gentamicin; Km, kanamycin; Sm, streptomycin; Nm, neomycin; Tc, tetracycline; Em, erythromycin; Cli, clindamycin; Cm, chloramphenicol; Am, ampicillin; PG, penicillin G; Van, vancomycin; Q-Da, quinupristin-dalfopristin; Lnz, linezolid; Tm, trimethoprim; Cip, ciprofloxacin; Rif, rifampicin.
[b]MIC values obtained with the MIC Test Strips system (MTS; Liofilchem).
[c]Broth microdilution breakpoints for coagulase negative staphylococci as established by the European Committee on Antimicrobial Susceptibility Testing (EUCAST) [33] and the Clinical and Laboratory Standards Institute for *Staphylococcus* spp. (CLSI) [34]; (-), cut-offs not established. Coloured, strains considered resistant by EUCAST (pink), CLSI (pale blue) or both (red). In bold, strains subject to genome sequencing.

Supplementary Table 2.- Genomic Distance Analysis of several *Staphylococcus* species type strains with the sequenced *Staphylococcus equorum* strains of this study. Results from digital DNA-DNA hybridization (dDDH) are shown below the self-comparison diagonal, and results from average nucleotide identity (OrthoANI) are shown above the diagonal.

ANI / dDDH	11A1I	16A1C	30A2I	50A2C	5A3I	48A3I	2A3C	8A3C	23A3C	CL10P	T17	1BCExtra	35A3C	*S. equorum* subsp. *equorum* NCTC 12414^T	*S. equorum* subsp. *linens* DSM 15097^T	*S. urealiyticus* DSM 6718^T	*S. casei* DSM 15096^T	*S. saprophyticus* ATCC 15305^T	*S. gallinarum* DSM 20610^T	*S. edaphicus* CCM 8730^T	*S. nepalensis* DSM 15150^T	*S. arlettae* NCTC 12413^T	*S. succinus* DSM 14617^T	*S. cohnii* NCTC 11041^T	*S. xylosus* CCM 2738^T	*S. kloosii* ATCC 43959^T	*S. pseudoxylosus* S04009^T
11A1I		94.7	99.9	94.8	97.4	94.8	94.7	94.8	94.7	94.6	94.7	94.6	94.8	94.8	94.7	78.7	80.4	80.2	77.4	79.2	79.2	76.0	80.1	79.1	79.7	75.7	80.2
16A1C	59.0		94.7	99.4	96.1	99.0	99.4	100	99.3	99.0	99.3	98.9	99.3	99.4	99.1	78.7	80.2	80.0	77.4	79.4	78.9	75.8	80.0	79.0	79.6	75.5	80.0
30A2I	99.2	59.0		94.7	97.4	94.8	94.8	94.8	94.7	94.6	94.6	94.5	94.9	94.8	94.8	78.7	80.4	80.0	77.4	79.1	78.9	75.7	80.1	79.3	79.6	75.7	80.1
50A2C	59.4	94.9	58.8		96.0	98.9	99.4	99.4	99.3	99.0	99.2	98.7	99.4	99.5	99.0	78.6	80.2	80.0	77.4	79.4	79.1	75.7	80.1	79.1	79.7	75.6	80.1
5A3I	77.4	66.0	77.3	66.5		95.9	95.8	96.0	95.9	95.7	95.9	96.2	95.8	95.9	96.0	78.6	80.1	80.3	77.5	79.3	79.1	75.9	80.2	79.2	79.7	75.9	79.9
48A3I	59.3	91.4	59.4	90.0	66.0		98.9	98.9	98.9	99.3	98.9	99.3	99.0	99.0	99.3	78.9	80.4	80.0	77.4	79.5	79.0	76.1	80.0	79.2	79.7	75.8	80.1
2A3C	59	94.4	58.8	95.4	65.3	90.3		99.3	99.4	99.1	99.5	98.9	99.5	99.4	99.2	78.7	80.2	79.7	77.7	79.4	78.9	75.9	80.1	79.1	79.6	75.7	80.2
8A3C	59.1	100	59.0	95.0	66.0	91.4	94.4		99.3	99.0	99.3	98.4	99.4	99.4	99.1	78.6	80.0	79.8	77.3	79.3	78.9	75.7	80.1	78.9	79.5	75.6	79.8
23A3C	59.1	94.0	59.0	93.6	65.5	90.4	94.7	94.0		99.0	99.8	98.7	99.8	99.4	99.0	78.7	80.2	80.1	77.5	79.4	79.2	75.9	80.1	79.0	79.8	75.7	79.9
CL10P	58.9	91.3	58.9	91.4	66.3	94.3	91.8	91.3	91.4		99.0	99.2	99.0	99.0	99.3	78.6	80.2	79.8	77.5	79.4	79.0	76.1	80.0	78.9	79.8	75.7	80.1
T17	59.1	94.2	59.3	94.1	65.7	90.8	95.2	94.2	98.6	95.1		98.7	99.7	99.5	99.0	78.8	80.2	80.1	77.4	79.4	78.9	76.0	80.1	79.3	79.6	75.7	70.9
1BCExtra	58.4	90.2	58.6	89.3	67.0	94.6	90.4	90.2	90.1	94.1	89.6		98.9	99.0	99.4	78.6	80.1	79.9	77.4	79.5	78.8	75.7	80.0	79.2	79.7	75.7	80.2
35A3C	59.5	94.7	59.5	94.9	66.0	91.1	95.7	94.7	98.6	91.6	98,7	90.3		99.5	99.2	78.6	80.3	80.0	77.3	79.4	79.0	75.9	80.2	79.0	79.6	75.7	80.0
NCTC 12414^T	59.3	95.6	59.3	96.4	66.0	91.9	95.5	95.6	96.0	91.9	95.8	91.6	95.9		-	-	-	-	-	-	-	-	-	-	-	-	-
DSM 15097^T	58.8	92.3	58.8	92.7	65.5	94.2	92.6	92.3	92.2	94.0	92.2	96.0	92.6	-		-	-	-	-	-	-	-	-	-	-	-	-
S. urealiyticus DSM 6718^T	22.8	22.8	22.7	22.6	22.7	22.8	22.7	22.8	22.6	22.6	22.9	22.8	22.9	-	-		-	-	-	-	-	-	-	-	-	-	-
S. casei DSM 15096^T	24.3	23.7	24.2	23.8	24.1	24.0	24.1	23.7	23.7	23.7	23.8	24.0	24.0	-	-	-		-	-	-	-	-	-	-	-	-	-
S. saprophyticus ATCC 15305^T	24.1	23.3	23.9	23.6	24.1	23.5	23.4	23.3	23.7	23.4	23.5	23.3	23.5	-	-	-	-		-	-	-	-	-	-	-	-	-
S. gallinarum DSM 20610^T	21.5	21.4	21.4	21.5	21.5	21.6	21.8	21.4	21.5	21.7	21.5	21.6	21.5	-	-	-	-	-		-	-	-	-	-	-	-	-
S. edaphicus CCM 8730^T	22.8	22.7	22.8	22.8	22.9	22.7	22.8	22.7	22.7	22.7	22.8	22.8	22.7	-	-	-	-	-	-		-	-	-	-	-	-	-
S. nepalensis DSM 15150^T	22.8	22.6	22.9	22.7	23.0	22.7	22.7	22.6	22.7	22.6	22.7	22.7	22.7	-	-	-	-	-	-	-		-	-	-	-	-	-
S. arlettae NCTC 12413^T	20.9	20.4	20.8	20.8	21.1	20.9	20.9	20.4	20.5	20.9	20.6	20.7	20.6	-	-	-	-	-	-	-	-		-	-	-	-	-
S. succinus DSM 14617^T	23.6	23.3	23.5	23.4	23.4	23.3	23.4	23.3	23.5	23.6	23.5	23.4	23.5	-	-	-	-	-	-	-	-	-		-	-	-	-
S. cohnii NCTC 11041^T	22.8	22.6	23.0	22.8	22.9	22.6	22.9	22.6	22.7	22.5	22.8	22.8	22.8	-	-	-	-	-	-	-	-	-	-		-	-	-
S. xylosus CCM 2738^T	23.3	23.2	23.2	23.4	23.4	23.2	23.4	23.2	23.2	23.3	23.3	23.2	23.3	-	-	-	-	-	-	-	-	-	-	-		-	-
S. kloosii ATCC 43959^T	20.5	20.2	20.5	20.2	20.4	20.3	20.3	20.2	20.3	20.3	20.4	20.4	20.4	-	-	-	-	-	-	-	-	-	-	-	-		-
S. pseudoxylosus S04009^T	23.4	23.4	23.4	23.4	23.5	23.4	23.5	23.4	23.4	23.6	23.4	23.4	23.4	-	-	-	-	-	-	-	-	-	-	-	-	-	

dDDH formula d4 (a.k.a. GGDC formula 2): sum of all identities found in HSPs divided by overall HSP length [67]. OrthoANI was calculated using the OrthoANIu algorithm, an improved iteration of the original algorithm and using USEARCH instead of BLAST [68]. Coloured boxes show the highest dDDH, and OrthoANI with the *S. equorum* type strains that are higher (orange and green) or lower (pale blue) than currently accepted thresholds for the species level (>70% and >95%, respectively). -, not done. In bold, dDDH and OrthoANI values for the strains sequenced in this work and the two *S. equorum* subspecies type strains: *S. equorum* subsp. *equorum* NCTC 12414^T and *S. equorum* subsp. *linens* DSM 15097^T.

References

18. Marty, E.; Bodenmann, C.; Buchs, J.; Hadorn, R.; Eugster-Meier, E.; Lacroix, C.; Meile, L. Prevalence ofantibiotic resistance in coagulase-negative staphylococci from spontaneously fermented meat productsand safety assessment for new starters. *Int. J. Food Microbiol*. **2012**, *159*, 74-83. doi: 10.1016/j.ijfoodmicro.2012.07.025.

33. EUCAST. Breakpoint tables for interpretation of MICs and zone diameters. Version 13.0, valid from 2023-01-01. https://www.eucast.org/clinical_breakpoints/, **2023**.

34. CLSI. Performance standards for antimicrobial susceptibility testing, 33rd ed. CLSI supplement M100. Clinical and Laboratory Standards Institute; **2023**.

43. Turnidge, J.; Kahlmeter, G.; Kronvall, G. Statistical characterisation of bacterial wild-type MIC value distributions and thedetermination of epidemiological cut-off values. *Clin. Microbiol. Infect*. **2006**, *12*, 418-25. doi: 10.1111/j.1469-0691.2006.01377.x.

67. Meier-Kolthoff, J.P.; Göker, M. TYGS is an automated high-throughput platform for state-of-the-art genome-based taxonomy. *Nat. Commun*. **201**9, *10*, 2182. doi: 10.1038/s41467-019-10210-3.

68. Yoon, S.H.; Ha, S.M.; Lim, J.M.; Kwon, S.J.; Chun, J. A large-scale evaluation of algorithms to calculate average nucleotide identity. *Antonie van Leeuwenhoek* **2017**, *110*, 1281-1286. doi: 10.1007/s10482-017-0844-4.
